# Oxytocin: a neglected hormone in pituitary disease - From function to the diagnosis of a deficiency, resulting clinical relevance, and potential treatment options in endocrinology

**DOI:** 10.20945/2359-4292-2025-0259

**Published:** 2025-11-24

**Authors:** Svenja Leibnitz, Mirjam Christ-Crain, Cihan Atila

**Affiliations:** 1 Department of Endocrinology, Diabetology and Metabolism, University Hospital Basel, Basel, Switzerland; 2 Department of Clinical Research, University of Basel, University Hospital Basel, Basel, Switzerland

**Keywords:** Oxytocin deficiency, posterior pituitary, hypopituitarism, AVP deficiency, neurophysin I

## Abstract

Oxytocin (OXT) is a neuropeptide hormone that plays a central role in numerous physiological and socio-emotional processes. Similar to arginine vasopressin (AVP), it is synthesized in the supraoptic and paraventricular hypothalamic nuclei and released both centrally and peripherally. Peripherally, OXT regulates uterine contractions during childbirth and milk ejection during lactation, metabolism, bone health, and cardiovascular functions. Centrally, it modulates social behavior, influencing trust, empathy, stress regulation, and emotional processing. Despite its close connection to AVP, the clinical significance of OXTDeficiency has only recently gained attention, particularly in patients with hypothalamic or pituitary damage with concomitant AVP-Deficiency. OXT-Deficiency may contribute to various neuropsychological symptoms seen in these patients, including social dysfunction, anxiety disorders, and reduced quality of life. However, a major challenge lies in accurately measuring OXT and thereby diagnosing a potential OXT-Deficiency. Basal plasma levels are unreliable, and most studied provocation tests only stimulate to a limited degree; hence, stronger provocation tests (e.g., using MDMA) and new surrogate parameters such as neurophysin I (NP-I) are gaining traction. Preliminary evidence from case reports and one small study suggests that intranasal OXT administration in patients with hypothalamic disorders may have beneficial effects on social behavior and emotion recognition. However, there is a clear need for larger, well-designed clinical trials, and several trials are currently underway to investigate the therapeutic potential of OXT in patients with AVP-Deficiency. OXT is also being explored as a possible treatment option in psychiatric conditions such as autism spectrum disorder, borderline personality disorder, and social anxiety disorder, with controversial results so far.

## OXYTOCIN - ANATOMY AND PHYSIOLOGY

### Synthesis, storage, and release

Oxytocin (OXT) plays a crucial role in both neurobiological and physiological functions, ranging from reproductive processes to the regulation of complex socio-emotional behaviors. OXT is closely related to arginine vasopressin (AVP), which primarily regulates fluid balance. Both hormones are synthesized by magnocellular and parvocellular neurons located in the paraventricular (PVN) and supraoptic (SON) nuclei of the hypothalamus (^1^,^2^). These neurons project to the pituitary gland for peripheral hormone release and also enable central release via dendritic diffusion and axonal projections (^3^). OXT and AVP are both nonapeptides, differing by only two amino acids at positions 3 and 8, and are encoded in close proximity on chromosome 20 in their precursor forms (^4^). OXT is initially synthesized as an inactive prepropeptide together with its carrier protein, neurophysin I (NP-I), which is essential for transport and storage.

The prohormone is then processed into the active peptide through cleavage and amidation (^1^,^5^). Both hormones signal through G protein-coupled receptors (V1AR, V1BR, V2R, OXTR), which show significant sequence homology and therefore allow cross-reactivity. The OXT receptor (OXTR) is widely distributed in the brain, including the hypothalamus, amygdala, anterior cingulate cortex, olfactory nucleus, and limbic areas (^6^,^7^). Notably, OXTR expression and density differ by sex (^8^) and vary across species, such as between monogamous and promiscuous voles (^9^). Peripherally, OXTRs are expressed in the uterus, ovaries, testes, mammary glands, kidneys, thymus, pancreas, adrenal glands, and adipose tissue (^4^). OXT concentrations in the brain are up to 1,000 times higher than in the blood, and its half-life in the central nervous system (CNS) is more than three times longer than in the periphery (19 *vs.* 6 minutes) (^10^-^12^).

### Peripheral function

**Figure 1:**
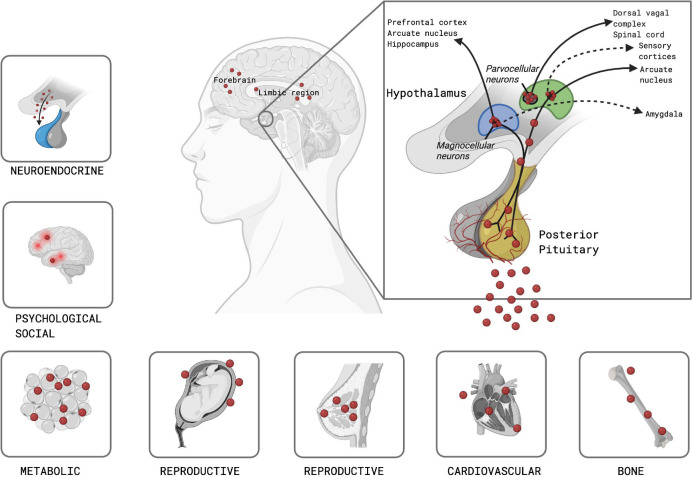
The hypothalamic-posterior-pituitary axis. Schematic representation of the neuroanatomical and functional organization of the hypothalamic-posterior-pituitary axis and its systemic effects. The hypothalamus receives input from multiple brain regions, including the limbic system, prefrontal cortex, hippocampus, sensory cortices, and amygdala, which converge on parvocellular and magnocellular neurons. These neurons control the release of the neuropeptides oxytocin and vasopressin into the circulation via the posterior pituitary. The lower panels illustrate the wide-ranging systemic effects of this axis, including neuroendocrine stress regulation, socio-emotional and behavioural modulation, metabolic balance, reproductive functions (such as uterine contraction and lactation), cardiovascular control, and bone health.

OXT is widely known for its role in human parturition and lactation, but it also exerts a broad range of physiological effects, including impacts on metabolism, bone health, the cardiovascular system, and psychosocial behavior (**Figure 1**). During pregnancy, uterine sensitivity to OXT increases significantly. Around the onset of labor, OXTR density rises up to 200-fold, facilitating uterine contractions (^13^,^14^). OXT also stimulates breast myoepithelial cells to initiate the milk let-down reflex during lactation. Infant suckling triggers further its release, promoting continued milk ejection (^13^,^14^). The peptide hormone also contributes to the regulation of metabolism. In rodents, central OXT administration reduces food intake and body weight, while increasing energy expenditure and brown fat thermogenesis (^10^). In normal-weight humans, intranasal OXT acutely improves glucose tolerance and beta-cell function, reduces both hungerand reward-driven eating in obese men, and lowers snack intake in both normal-weight and obese individuals (^15^,^16^). Additionally, OXTRs are expressed in both osteoblasts and osteoclasts (^17^), and OXT has anabolic effects on bone, promotes ossification, and inhibits bone resorption (^17^-^19^). Clinically, low OXT levels have been associated with reduced bone mineral density (BMD) in patients with AVP-Deficiency (^20^). Moreover, OXT has been implicated in the modulation of atherosclerotic processes through its anti-inflammatory and antioxidative properties (^21^,^22^). In addition, its ability to reduce food intake, body weight gain, insulin resistance, and to regulate the hypothalamic-pituitaryadrenal (HPA) axis contributes to its potential cardioprotective effects (^23^).

Figures created with biorender.com.

### Social and behavioral function

**Figure 2:**
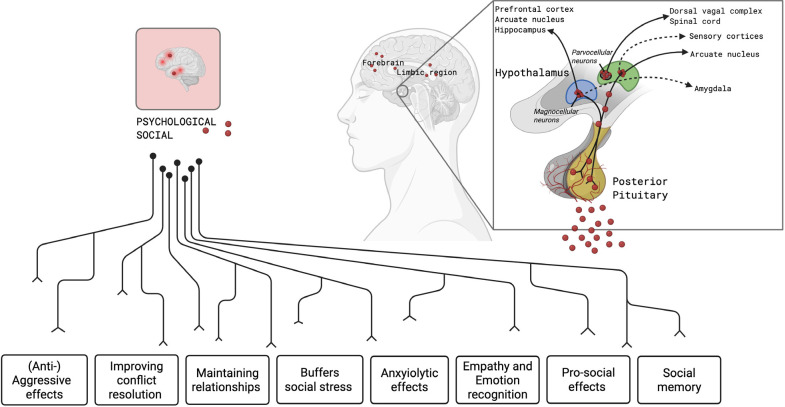
Psychosocial and emotional functions of central oxytocin signalling. Oxytocin is synthesized by parvocellular and magnocellular neurons in the hypothalamus and released via the posterior pituitary. These neurons receive inputs from key limbic and cortical regions linking oxytocin signalling to emotional and social processing. The left portion of the figure illustrates the diverse psychological and social effects of central oxytocinergic signalling, including: (anti-)aggressive effects, conflict resolution enhancement, relationship maintenance, buffering of social stress, anxiolytic (anxiety-reducing) effects, empathy and emotion recognition, promotion of pro-social behaviours, social memory consolidation.

OXT is increasingly recognized for its key role in regulating complex social-emotional and behavioral functioning (**Figure 2**) (^24^-^28^). In animal studies, central OXT administration increases partner preference in female prairie voles and promotes maternal behavior in female rats (^29^,^30^). Centrally given OXT also induces anxiolytic-like effects in rodents (^31^), partly mediated by the HPA axis (^32^,^33^), and intranasal OXT reduces aggressive behavior while promoting affiliation in male rats and mice (^34^,^35^). In humans, intranasal OXT enhances pro-social behaviors, including trust, intimacy, attachment, empathy, and emotion recognition (^27^,^28^,^36^,^37^). It also facilitates the formation of social memories and may enhance self-perception (^38^). Additionally, the anxiolytic effects of OXT are well established: It buffers responses to social stress, lowers cortisol levels during conflicts (^39^), decreases amygdala activity, and increases insular cortex activation in response to emotional stimuli (^28^,^40^), suggesting that OXT strengthens the stress-buffering role of social support (^41^). Notably, study participants receiving both social support and OXT exhibited the lowest cortisol levels during social stress (^24^). Similarly, endogenous OXT release during breastfeeding was associated with reduced pituitary-adrenal responses to psychosocial stress in postpartum women (^42^).

Figures created with biorender.com.

### Exogenous administration of oxytocin

Because OXT is rapidly metabolized by chymotrypsin in the gut, oral administration is not possible. Intranasal delivery is therefore a practical and well-tolerated alternative, approved to support milk let-down in breastfeeding women, while intravenous OXT is commonly used to induce uterine contractions during labor. In research, intranasal OXT is typically administered in doses ranging from 6 to 48 IU, with 24 IU being the most commonly used dose in human behavioral studies (^40^,^43^). Importantly, these studies do not address OXT treatment for a clinically diagnosed deficiency, as no such condition has yet been established with defined diagnostic criteria. Rather, they focus on the experimental administration of OXT in healthy participants.

Growing evidence indicates that intranasal application can produce functionally relevant central effects. The intranasal route largely bypasses the blood-brain barrier and allows efficient central delivery, with peak effects, particularly in amygdala reactivity, observed approximately 45 minutes after administration (^44^). For instance, studies show that 24 IU reduces amygdala responses to fear and improves recognition of ambiguous faces (^44^). Consistent with these findings, several trials report that standard intranasal doses enhance emotion recognition, promote empathy, and increase trust in context-dependent ways (^37^,^44^-^46^). Together, these observations point to the potential of peripheral administration to influence central mechanisms. Peak plasma levels usually occur 10 to 90 minutes after intranasal application (typically 24-30 IU) (^47^,^48^), while maximum cerebrospinal fluid concentrations are reached up to 75 minutes post-administration (^49^,^50^). Intravenous OXT is commonly administered to induce or augment labor and has a short plasma half-life of 3-6 minutes (^51^), reaching steady-state levels within 40 minutes and rapidly returning to baseline after cessation of infusion. Due to the typically low infusion doses used during labor (often totaling 5-10 IU), OXT does not significantly cross the placenta or the blood-brain barrier (^52^).

## SYMPTOMS RELATED TO OXYTOCIN DEFICIENCY

### Changes in psychosocial, emotional, and eating behaviour

Hypopituitarism (HPD) is a condition with an estimated prevalence of 21 to 42 cases per million, characterized by partial or complete deficiency of one or more pituitary hormones, resulting from damage or dysfunction of the pituitary gland or hypothalamus (^53^-^55^). Deficiencies of anterior pituitary hormones are well recognized, with established clinical implications and standardized guidelines for hormone replacement (^53^). Similarly, deficiency of the posterior pituitary hormone AVP (formerly known as central diabetes insipidus) is well-characterized and treated with desmopressin (^56^). However, despite OXT being produced alongside AVP and released both centrally and peripherally via the posterior pituitary, its potential deficiency and clinical relevance have only recently begun to be explored. Disruptions of the vasopressinergic neurons causing AVP-Deficiency could potentially result in an impairment of oxytocinergic neurons and thereby causing an additional OXTDeficiency due close proximity of both systems (^57^). Hence, it is plausible to assume that a concomitant accompanying OXT-Deficiency is partly responsible for the increased psychopathology and decreased quality of life (QoL) seen in AVP-deficient patients (^57^).

HPD is associated with comorbidities such as sexual dysfunction, neuropsychological symptoms, altered eating behaviours, obesity, metabolic disorders, and osteoporosis (^58^-^63^). Specifically, HPD is linked to greater psychopathology, including increased severity of anxiety, depression, and alexithymia (difficulty identifying and expressing emotions). Patients with HPD also experience social dysfunction, impulsive behaviours, and hyperphagia, contributing to obesity and reduced QoL.

Recent research suggests a potential OXTdeficient state in HPD, particularly in patients with hypothalamic-posterior-pituitary damage. Given the anatomical location of hypothalamic OXT-producing neurons, patients with hypothalamic-pituitary dysfunction who develop AVP-Deficiency (^64^), often due to suprasellar lesions such as craniopharyngiomas (CP) and germinomas or following hypothalamicpituitary surgery, are presumably at the highest risk for co-existing OXT-Deficiency (^65^-^70^). Other causes include infiltrative or malignant diseases, neurosurgery, radiotherapy, head trauma, and congenital abnormalities. Early findings indicate that damage to OXT neurons may contribute to symptoms of HPD, including emotional dysregulation, social dysfunction, and metabolic disturbances (^65^-^70^). Therefore, given OXT’s role in modulating mood, social behaviour, impulse control, and eating habits, a deficiency in OXT signalling may contribute to these symptoms observed in HPD. However, particularly in CP patients, these symptoms may also result directly from hypothalamic injury, whether caused by the tumor or secondary to surgical intervention.

More than half of individuals treated for sellar tumours develop hyperphagic obesity, which is associated with reduced functional capacity, metabolic syndrome, cardiovascular disease, and increased mortality (^15^,^71^). Hypothalamic damage, particularly in the PVN, disrupts energy homeostasis, leading to excessive hunger and reduced energy expenditure (^72^-^75^). The extent of hypothalamic damage and presence of AVP-Deficiency are predictors of hypothalamic obesity (^74^,^76^,^77^). Many studies have focused on CP, a condition associated with a high risk of causing damage to vasopressinergic and oxytocinergic neurons (^78^). A long-term study of 10 years identified changes in personality and social deficits (^75^,^78^-^80^). While desmopressin therapy improves clinical outcomes, there is evidence indicating that patients still may not attain population norms in terms of QoL and socio-emotional abilities (^81^-^83^). Data from the so-far largest survey study in patients with AVP-Deficiency showed a high prevalence of self-reported psychological problems: 25% reported heightened anxiety, 25% sleep disturbances, 23% depressed mood, 18% stress management disturbance, and 12% personality change (^84^). In total, 64% reported lower QoL affecting recreation and fun, social activities, and both physical and mental well-being (^84^). This was observed in patients with and without additional anterior pituitary hormone dysfunctions, which challenges the notion that reduced anterior pituitary hormone dysfunctions are the primary cause of psychological changes. Importantly, adult patients with growth hormone (GH) deficiency are frequently neither evaluated for nor treated with GH replacement therapy, which may contribute to reduced QoL and should be considered alongside potential effects of presumed OXT-Deficiency.

However, while patients with AVP-Deficiency are presumed to be at the highest risk for OXT-Deficiency, not all individuals with AVP-Deficiency necessarily experience OXT-Deficiency. Some may retain or regenerate OXT-producing neurons, particularly in milder cases. Importantly, recent studies also highlight higher ACTH and cortisol response to stress in patients with AVP-Deficiency compared to controls, indicating impaired HPA axis regulation (^85^,^86^). Among other mechanisms, co-existing OXT-Deficiency in these patients may contribute to elevated cortisol levels, as OXT is known to modulate stress responses and attenuate HPA axis activation.

### Changes & complications in obstetric functions

OXT has well-known obstetric functions (^87^,^88^). The current data are limited to case series involving patients with hypopituitarism, and there has been insufficient investigation on difficulties with breastfeeding or complications during labour in patients with AVPDeficiency (^89^-^93^). Within these cases, some patients reported successful spontaneous labour without the need for OXT administration (^89^,^90^). This aligns with the observation that, at hospital discharge, only half of the patients were breastfeeding, implying that OXT from the pituitary may not be mandatory or could be partially preserved for the initiation of spontaneous labour or milk let-down (^93^-^95^).

## UNMASKING AN OXYTOCIN DEFICIENCY

Few initial studies measured OXT in patients with HPD and mainly focused on basal measurements (^65^,^69^,^96^,^97^). One of the first studies assessed fasting and postprandial saliva OXT in long-term CP survivors and healthy controls, with no noteworthy difference between the groups. Contrary to these results, another study in patients with hypopituitarism compared to healthy controls demonstrated lower basal OXT levels regardless of the presence of AVP-Deficiency (^97^). The lower OXT level was also associated with impaired ability to identify facial expressions accurately (^97^). In a study that focused on male patients with AVP-Deficiency, symptoms and signs of depression, anxiety, and alexithymia were greater compared to both patients with hypopituitarism but without AVPDeficiency and healthy controls (^98^). Basal OXT levels were similar in all groups; however, patients with AVPDeficiency had a slightly lower level when plasma OXT was pooled over an hour (^98^). On the contrary, Eisenberg and cols. noted increased basal OXT levels in patients compared to healthy controls, once again raising doubts about the reliability of basal OXT levels as an indicator of a potential deficiency (^96^,^99^).

These controversial findings might partly be explained by difficulties in accurately measuring OXT, given its technical complexities, and ideal sampling methods are currently the subject of research (see below) (^100^,^101^). Importantly, as indicated by a metaanalysis, central levels of OXT correlate with peripheral levels only following stimulation, not at baseline (^102^). Therefore, a provocation test is crucial in assessing a possible OXT-Deficiency. Unlike other endocrine axes, there is no established “normal” reference range for OXT in any biological fluid, and its pulsatile secretion means that a single measurement may not accurately reflect OXT status (^103^). Moreover, central and peripheral OXT release do not always align, further complicating interpretation. To overcome these challenges, a provocative test, similar to those used for adrenal insufficiency and growth hormone deficiency, is needed to unmask OXT-Deficiency. Various provocation tests have already been examined for OXT (**Table 1**). Overall, the weak and clinically irrelevant OXT stimulation is considered insufficient for diagnosing OXT-Deficiency. Supraphysiological stimulation is required for a valid provocation test, and psychoactive stimulation is necessary to assess the central action.

**Table 1:** Selected oxytocin provocation tests in humans

Provocation Test^*^	Measurement	Participants	Effect on Oxytocin	Findings on psychological endpoints	Limitations
MDMA (^110^,^168^)	Plasma OXT	Healthy controls Patients with AVP-D	500%-800% increase in healthy controls. No increase in patients.	Under stimulation the typical prosocial, anxiolytic and positive effects of MDMA in healthy controls. No relevant or reduced psychoactive effects in patients under MDMA.	Cardiovascular side effects. Restriction to use in clinical practice.
Ethinyloestradiol or Oestradiol or physiological menstrual cycle (^169^-^171^)	Plasma OXT	Healthy controls Female patients undergoing reproductive stimulation	100%-400% increase in healthy controls.	N/A	High doses of Oestrogen-derivates have the risk of thromboembolism.
Insulin Tolerance Test (^104^,^170^,^172^)	Plasma OXT	Healthy controls Patients with AVP-D	100% increase in healthy controls. No increase in patients.	N/A	Test specific side effects and patient discomfort *(e.g,* hypoglycaemia-induced symptoms). Contraindications in common populations.
Physical Exercise or sexual self stimulation (^69^,^70^,^173^)	Salivary OXT	Healthy controls Patients craniopharyngioma	50%-200% increase in healthy controls. No OXT increase in patients.	Patients showed lower empathy and emotional recognition, higher depression and anxiety scores, and higher self-reported autistic traits compared to healthy controls.	Might show high variability and lack reproducibility.
Psychosocial Stress (TSST) (^173^,^174^)	Plasma/Salivary OXT	Healthy controls	50%-150% increase in healthy controls.	Higher stress-induced oxytocin secretion is associated with greater cortisol reactivity during the stressor, followed by parasympathetic recovery in the post-stress phase.	Might show high variability and lack reproducibility.
Hypertonic Saline (^108^)	Plasma OXT	Healthy controls		N/A	Test specific side effects *(e.g.,* hypernatremia, strong thirst, general malaise). No sufficient increase to be administered as a diagnostic stimulation test.
Kisspeptin (^109^)	Plasma OXT	Healthy controls	20% increase from baseline.	N/A	Not established stimulation test. Increase only observed in men. No sufficient increase to be administered as a diagnostic stimulation test.
Glucagon (^105^)	Plasma OXT	Healthy controls Patients with AVP-D	20% increase from baseline in both groups	N/A	Test specific side effects and patient discomfort. No sufficient increase to be administered as a diagnostic stimulation test.
Melatonin (^107^)	Plasma OXT	Healthy controls Patients with hypopituitarism	20% increase from baseline in healthy controls. No increase in patients.	Greater depression symptoms, alexithymia, impaired sexual function and worse QoL in patients compared to healthy controls.	No sufficient increase to be administered as a diagnostic stimulation test.
Corticotropin-Releasing Hormone (^106^)	Plasma OXT	Healthy controls Patients with hypopituitarism	No major effect	Greater psychopathology, sexual dysfunction and worse QoL in patients compared to healthy controls.	No sufficient increase to be administered as a diagnostic stimulation test. Currently not available as a stimulation test.
Arginine (^108^)	Plasma OXT	Healthy controls	No major effect	N/A	Test specific side effects and patient discomfort *(e.g,* nausea). No sufficient increase to be administered as a diagnostic stimulation test.
Macimorelin (^108^)	Plasma OXT	Healthy controls	No major effect	N/A	No sufficient increase to be administered as a diagnostic stimulation test.

* Listed studies are only provided as explanatory examples. Abbreviations: MDMA = 3,4-Methylenedioxymethamphetamine; TSST = Trier Social Stress Test; OXT = Oxytocin; N/A = Not applicable; AVP-D = Arginine Vasopressin Deficiency (formerly central diabetes insipidus).

Copyright® AE&M all rights reserved.

To this purpose, MDMA (3,4-methylenedioxymethamphetamine, ‘ecstasy’) was recently used as a stimulus for OXT (^110^). A single 100 mg dose resulted in an eightfold increase in plasma OXT in healthy adults but had no effect in patients with AVP-Deficiency. This striking differentiation makes MDMA a strong candidate for OXT-Deficiency testing. However, its potential side effects (*e.g.*, cardiovascular) and regulatory restrictions limit its widespread use. Lower doses may be explored as a safer alternative. MDMA increases peripheral OXT levels and central OXT-mediated behavioural effects associated with its empathic and prosocial profile. These effects include increased closeness and openness to others, enhanced trust, elevated happiness, and an overall sense of well-being (^111^-^114^). Basal plasma OXT levels showed no difference in patients with AVP-Deficiency and matched healthy controls. In response to MDMA, in controls, plasma OXT increased 8-fold, without a response in patients. In patients, psychoactive effects induced by MDMA (*e.g*., “good effect”, “liking effect” “feeling high”) were either blunted or absent compared to controls. Both patients and healthy controls showed a comparable increase in state anxiety at the start of the experiment. While anxiety levels decreased in the controls at the peak OXT concentration, the patients experienced no anxiolytic effect. The strong rise and subsequent action of OXT in central key areas linked to fear processing, like the amygdala, may be responsible for the anxiolytic effects seen during MDMA stimulation. Supporting this observation, the *Facial Emotion Recognition Task* showed decreased recognition of negative emotions (such as “fearful”) in healthy controls but not in patients. Notably, half of the patients exhibited clinically relevant anxiety and symptoms of emotional blindness, even though this study only included patients without actively treated psychological co-morbidities. Taken together, these results demonstrate that, in contrast to the predicted effects in controls, nearly all the psychoactive effects of MDMA were either blunted or absent in patients. This pattern is indicative of the OXT’s absence and the ensuing dysfunction in key brain areas essential for socioemotional processing.

However, not all patients, particularly those with cardiovascular conditions, are suitable candidates for MDMA stimulation, and its implementation into clinical routine poses challenges (^115^). The proposed use of MDMA as a diagnostic tool raises important considerations of tolerance, interindividual variability, and ethics (^116^). Evidence from clinical trials in post-traumatic stress disorder (PTSD) shows no development of tolerance or loss of efficacy with the limited short-course dosing regimens typically used (^117^,^118^). Nonetheless, individual variability should be considered. Regarding safety, adverse events are usually mild and transient, with serious complications rarely reported, yet comprehensive long-term safety data are lacking. Ethically, it is important to emphasize that, as of September 2025, MDMA-assisted therapy is not FDA-approved for PTSD (^119^). Although it received *Breakthrough Therapy* designation in 2017, the FDA approval is pending, and MDMA use remains restricted to controlled clinical research settings (^120^). Thus, application of MDMA in diagnostics outside trials is currently not permissible. Therefore, future research should focus on developing alternative, simplified approaches. One possibility may be to mimic the action of MDMA using substances that have already obtained approval (^121^,^122^).

An additional key barrier to identifying OXTDeficiency is the lack of standardized and validated OXT measurements. due to methodological and biological complexities (^99^,^123^-^126^). A key challenge is the unclear relationship between central and peripheral release as only few human studies have assessed OXT at baseline or in response to stimuli (^127^-^130^). According to a meta-analysis, no correlation between cerebrospinal fluid (CSF) and plasma levels was found at baseline, though a positive correlation appeared after stress in non-human species (^102^). Thus, peripheral levels do not reliably reflect central activity at baseline but in response to stimuli. Biological sex and age influence OXT (^131^,^132^); in women, the menstrual cycle, menopause, and hormonal therapy add variability (^133^). Lifestyle factors such as exercise, smoking, food, alcohol, nicotine, or caffeine show little association when proper extraction is used (^131^). The variability in replication arises from inconsistent extraction, different biological matrices, and diverse assays, with little correlation between measurement techniques (^123^,^134^-^136^). Standardization and cross-laboratory harmonization are therefore critical. Preanalytical factors also matter: fasting status, stress, tube type (*e.g.*, EDTA with inhibitors), centrifugation, storage, and freezing delays should be documented. Another complication is the pulsatile nature of OXT, which means that repeated or dynamic sampling is preferable (^102^,^103^,^137^,^138^). Common analytic methods include radioimmunoassay (RIA), enzyme-linked immunoassay (EIA/ELISA), and liquid chromatography-mass spectrometry (LC-MS). All require validation for specificity, precision, recovery, parallelism, and stability (^139^-^141^). A major debate concerns extraction, which removes interference and enriches peptide concentrations. Without extraction, immunoassays yield inflated results (^136^,^142^-^144^). A meta-analysis illustrates the impact: blood (unextracted ≈ 275.61 pg/mL *vs.* extracted ≈ 4.75 pg/mL), saliva (4.92 *vs.* 3.15 pg/mL), urine (47.42 *vs.* 13.20 pg/mL), and CSF (17.31 *vs.* 17.29 pg/mL) (^131^). These findings confirm that extraction is essential for plasma and urine, less so for saliva, and negligible for CSF (^136^).

Measurement of NP-I could help address some of the problems in OXT measurement. NP-I, the carrier protein co-released with OXT, has potential as a biomarker for assessing endogenous OXT secretion (^145^). Since it is secreted in equimolar concentrations to OXT and has a longer half-life, it may provide a more stable and reliable measure of OXT release over time. Unlike OXT, which is rapidly degraded by aminopeptidases and fluctuates in response to various stimuli, NP-I may offer a less transient readout of OXT dynamics. Previous studies, including pregnant women or estrogen administration, indicated strong correlations between NP-I and OXT (^100^,^146^-^149^). However, validation under acute OXT stimulation conditions is lacking. A recent secondary study using MDMA demonstrated an 8-fold increase in OXT and a 20-fold increase in NP-I in healthy participants, while patients exhibited only minimal changes in both biomarkers (^149^). NP-I levels strongly correlated with OXT (R = 0.92) and with subjective psychosocial effects, such as increased trust and reduced fear. Additionally, NP-I demonstrated superior analytical performance, requiring no complex extraction steps and reducing measurement time from 16-24 hours for OXT to under two hours (^101^). These findings establish NP-I as a robust and practical biomarker for assessing OXT function, comparable to how copeptin serves as a surrogate for AVP (^150^). By overcoming the limitations of direct OXT measurement, NP-I opens new avenues for research into conditions such as autism spectrum disorder, anxiety, and depression, offering deeper insights into their pathophysiology and potential therapeutic targets. NP-I could serve as a valuable surrogate marker for studying OXT-Deficiency and the response to OXT-based therapies in conditions such as hypopituitarism and neuropsychiatric disorders.

## OXYTOCIN REPLACEMENT THERAPY IN PATIENTS WITH AVP-DEFICIENCY

**Figure 3:**
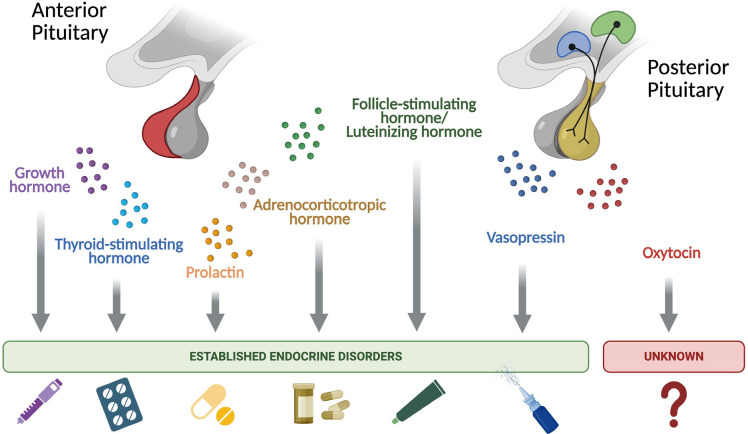
Established *vs.* unexplored endocrine disorders of the pituitary hormones. Overview of key hormones secreted by the anterior and posterior pituitary glands and their clinical relevance. Anterior pituitary hormones, including growth hormone, thyroid-stimulating hormone, prolactin, adrenocorticotropic hormone, and the gonadotropins, are well-characterized and their deficiency or excess is linked to clearly defined endocrine disorders, each with established diagnostic and therapeutic pathways. The posterior pituitary secretes vasopressin, whose deficiency results in a well-defined clinical syndrome with clear treatment options. In contrast, although oxytocin is similarly secreted by the posterior pituitary, its deficiency is not linked to a recognized disease state in humans, and no formal diagnostic criteria or replacement therapies currently exist.

To date, no preclinical studies have investigated the effects of OXT on socio-emotional behavior in AVPdeficient animal models. Moreover, clinical evidence remains scarce (**Figure 3**). A systematic review of the OXT system in patients with CP, a condition at high risk of developing AVP-Deficiency (^78^), identified only two case reports describing long-term low-dose intranasal OXT administration and one pilot study assessing the acute effects of a single OXT dose on emotion recognition (^66^,^67^,^151^,^152^).

Figures created with biorender.com.

One case report described a 6-year-old girl with panhypopituitarism and hypothalamic damage following CP resection, who developed socioemotional dysfunction (^67^). Treatment with 4 IU/day intranasal OXT was associated with improved social engagement and family bonding. Another case involved a 13-year-old boy with hypothalamic obesity post-CP resection, treated with 6 IU/day OXT for 10 weeks, followed by combined therapy with naltrexone (100 mg/day) for an additional 38 weeks (^152^). This regimen led to reductions in BMI and hyperphagia, suggesting that OXT may modulate reward-driven eating through HPA axis regulation and glucose metabolism. In a pilot cross-sectional study of 10 patients with childhoodonset CP (nine of the patients having AVP-Deficiency), a single 24 IU intranasal OXT dose increased salivary and urinary OXT levels and improved recognition of negative emotions (^66^). However, the small sample size and absence of placebo control limit the strength of conclusions regarding the therapeutic potential role of OXT in patients with AVP-Deficiency. Considering the high prevalence of psychosocial comorbidities and neurobehavioral dysfunction in this patient population, potentially linked to underlying OXT-Deficiency (^69^,^96^,^98^), there is a clear need for larger randomized controlled trials.

In this line, three randomized, double-blind, placebo-controlled trials are currently being conducted at the University Hospital of Basel in Switzerland to investigate intranasal OXT as a novel treatment for psychological symptoms and socio-emotional dysfunction in AVP-Deficiency. The largest of these is a multicenter, parallel-arm study enrolling 112 AVP-deficient patients, who are randomly assigned to receive either intranasal OXT (24 IU twice daily) or placebo over a 28day period. Primary outcomes include changes in anxiety and emotion recognition, assessed through validated questionnaires and computerized empathy and emotion recognition tasks. In addition, two smaller cross-over studies, each involving 21 AVPdeficient patients and matched healthy controls, are investigating the effects of intranasal OXT (24 IU once daily) versus placebo. One study focuses on the recognition of facial emotions and body expressions, as well as physiological and psychological responses to acute social stress, while the other study examines the impact of OXT on sexual well-being and intimacy. Finally, a further diagnostic study aims to evaluate whether low-dose MDMA (25 or 50 mg) can safely stimulate OXT release in AVP-deficient patients, potentially offering a novel diagnostic tool for assessing OXT-Deficiency. The outcomes of these trials may have the potential for a paradigm shift in clinical management of AVP-Deficiency.

## OXYTOCIN TREATMENT IN OTHER DISORDERS

Over recent decades, evidence has suggested that OXT function might be impaired in mental disorders associated with social deficits (^153^,^154^). Intranasal OXT has recently gained attention as a potential treatment for these conditions, but findings, especially in autism spectrum disorder (ASD), remain mixed and sometimes controversial (^155^-^158^). In borderline personality disorder, intranasal OXT has been shown to enhance empathy and social approach, particularly by improving responses to negative social cues (^159^). In generalized social anxiety disorder, OXT reduces amygdala hyperactivity in response to threatening social stimuli, as demonstrated in functional MRI studies (^160^). In ASD, where social communication deficits are a core feature, clinical trials have shown inconsistent results. One randomized controlled trial (RCT) in male youth with ASD showed improved performance on the *Reading the Mind in the Eyes Task* with OXT compared to placebo (^161^). However, a large 24-week placebo-controlled trial found no significant effects of OXT on social or cognitive outcomes (^162^). A metaanalysis of 28 studies reported some positive effects on social functioning, but no consistent improvements in non-social symptoms (^158^). Overall, heterogeneity in study designs, dosages, participant characteristics, and symptom profiles complicates comparisons across trials (^162^). While OXT may promote prosocial behavior, its effectiveness across all core ASD symptoms remains uncertain (^156^). Importantly, unlike AVP-Deficiency, no primary OXT-Deficiency per se has been identified in these disorders. Current evidence is largely observational, linking variations in social behavior to alterations in peripheral OXT levels (^163^), genetic differences in OXT signaling pathways (^164^), or OXT receptor expression (^165^).

Evidence from diet-induced obese animal models has shown that OXT administration reduces food intake, increases energy expenditure, and leads to body weight reduction (^10^). Therefore, OXT has recently been proposed as a promising treatment option for obesity. Although a pilot trial in adults with obesity demonstrated a clinically relevant BMI reduction (^166^), a recent placebocontrolled study found that intranasal OXT, administered four times daily for eight weeks in adults with obesity, did not reduce body weight or improve body composition (^167^).

## CONCLUSION AND OUTLOOK

OXT is far more than a “birth hormone” - its central role in emotional, social, and metabolic regulation makes it a compelling target for clinical research and therapy. While the pathophysiological foundations and therapeutic potential of OXT-Deficiency in patients with AVP-Deficiency are becoming increasingly clear, standardized diagnostic procedures and robust clinical evidence are still lacking. The use of NP-I as a surrogate marker, along with promising results from MDMA-based provocation testing, may pave the way for more accurate diagnostics. Ongoing clinical trials on OXT replacement therapy in patients with AVPDeficiency have the potential to transform current treatment approaches. Meanwhile, research on OXT in psychiatric disorders shows promise but remains heterogeneous. Overall, there is a strong need for further well-controlled studies to clarify the diagnostic and therapeutic role of OXT in both endocrinological and neuropsychiatric contexts.

## Data Availability

datasets related to this article will be available upon request to the corresponding author.
